# Application of a real-time cell analysis system in the process development and quantification of Rift Valley fever virus clone 13

**DOI:** 10.1099/acmi.0.000191

**Published:** 2020-12-17

**Authors:** Boitumelo Moetlhoa, Leeann Naicker, Rose Hayeshi, Anne Grobler, Nobalanda B. Mokoena, Crispen Mawadza

**Affiliations:** ^1^​ Onderstepoort Biological Products (OBP) SOC Ltd, 100 Old Soutpan Road, Onderstepoort 0110, South Africa; ^2^​ DST-NWU Preclinical Drug Development Platform (PCDDP), North-West University, Potchefstroom 2520, South Africa

**Keywords:** cell index, real-time cell analysis, Rift Valley fever, roller bottle system, viral plaque assay

## Abstract

Conventional cell-culture viral quantification methods, namely viral plaque and 50 % tissue culture infective dose assays, are time-consuming, subjective and are not suitable for routine testing. The viral plaque formation assay is the main method utilized for Rift Valley fever virus (RVFV) clone 13 quantification. The RVFV is a mosquito-borne RNA *Phlebovirus* belonging to the family *Bunyaviridae*. The virus comprises a single serotype and causes the zoonotic Rift Valley fever disease. The real-time cell analysis (RTCA) system has been developed for the monitoring of cell growth, cell adhesion, cell viability and mortality using electronic impedance technology. In this study, Vero cell growth kinetics and RVFV clone 13 replication kinetics were investigated in a roller bottle and RTCA systems. In roller bottles, Vero cell growth was measured by cell counts through trypan blue staining, whilst impedance expressed as the cell index (CI) was used for Vero growth measurement in the RTCA system. Similar growth patterns were observed in both roller bottle and RTCA systems. Exponential growth phase was observed between 48 and 100 h, followed by a stationary phase from 100 to 120 h, before cell death was observed. Viral plaque assay quantification of RVFV clone 13 in the roller bottle system and the time required for the CI to decrease 50 % after virus infection (CIT50) in the RTCA system were comparable. The highest RVFV clone 13 titre was obtained at 120 h in both roller bottle and RTCA systems. An increase in time for cytopathic effect (CPE) formation was observed with a decrease in the concentration of the virus used to infect the RTCA plates. A positive correlation was observed between the viral concentration and the time for a CPE and was used to calculate CIT50. A similar correlation was observed between the viral concentration and the time for a CPE in the roller bottle system. This study shows that the RTCA system can be used as an alternative method for conducting cell culture kinetics and viral quantification.

## Introduction

Rift Valley fever is a zoonotic mosquito-borne viral disease that results from an infection with Rift Valley fever virus (RVFV). This virus belongs to the genus *Phlebovirus* within the family *Bunyaviridae* and comprises a single serotype [1–5]. The RVFV is an RNA virus comprising a three segment genome, namely L, M and S. The L and M segments are of negative polarity and encode RNA polymerase and the glycoprotein precursor, respectively. The S segment is of ambisense polarity and encodes the nucleoprotein N and non-structural protein NSs [2, 4, 6, 7]. The negative-sense RNA genome is transcribed by an RNA polymerase before translation can occur [4, 6]. During the viral replication cycle of RVFV, the glycoproteins are known to play a vital role in the penetration, maturation and budding processes of the virion through the use of receptors that have not yet been identified [8].

Various measures are carried out to reduce the loss of livestock caused by Rift Valley fever, including vaccination, vector control by biological or chemical means or the movement of livestock from low-situated swampy areas to higher areas during seasons of heavy rainfall [9]. Vaccination of livestock against viral diseases has been found to be the most sustainable strategy utilized to date to mitigate the impact of these viruses on livestock and agriculture.

Determination of viral infectious titres is essential during process development for vaccine production [10]. RVFV replicates well in *Aedes* species mosquitoes and mammalian cell cultures. *In vitro* viral quantifications are conducted by plaque or 50 % tissue culture infective dose (TCID_50_) assays, by measuring cytopathic effect (CPE) on monolayers of African green monkey kidney (Vero) cells, which is very time consuming [11, 12]. Utilization of adherent cell lines in vaccine production has grown since its introduction in the 1970s, and currently the science of these cell lines has branched from vaccine production to other therapeutic products such as antibodies [13]. Various cell lines have become essential biological tools in research for animal and human virus isolation, characterization and preparation of live attenuated viral vaccines *in vitro* [14–16].

The real-time cell analysis (RTCA) system has been developed for the monitoring of cell growth, cell adhesion, cell viability and mortality using electronic impedance technology [15, 17, 18]. The impedance detected by the system analyser in wells of growing cultures is reported as the cell index (CI). The CI gives a measure of parameters such as cell size, cell–substrate or cell–cell attachment. The RTCA provides the real-time monitoring of cell growth and death, compared to end-point measurements. The attachment and spread of cells across the sensor surface of an electrode increases impedance and, therefore, CI recorded. When cells round up, detach or are infected with viruses, the impedance value and, therefore, CI drops. This does not directly define the CI as a direct reflection of cell count, but as a collective mode of attachment, cell membrane composition or metabolic state of the cell [18, 19].

In this study, the RTCA system was evaluated as a comparative method to the plaque forming assay for determining RVFV clone 13 titre. The system was also utilized to determine Vero cell culture kinetics in comparison with the conventional roller bottle scale system. The comparative data suggest the RTCA system can be utilized for determination of process parameters during upscaling.

## Methods

### Cells and virus

Vero adherent cells used in this study were purchased from the European Collection of Authenticated Cell Cultures (ECACC, UK). The cells were cultured and maintained in 850 cm^2^ roller bottles at 37±2 °C in Glasgow minimum essential medium (GMEM; Gibco), supplemented with 10 % (v/v) bovine serum (Starred), 6.7 µg amphotericin B ml^−1^ (Oxoid) and 0.1 g streptomycin l^−1^ . The cells were sub-cultured every 5 days and were counted using a TC10 automated cell counter (Bio-Rad) after staining with trypan blue 0.2 % [20]. The RVFV clone 13 strain, 74HB59, was obtained from Onderstepoort Biological Products (OBP) seedstock inventory following a standard operating procedure (OBP). The virus was amplified in Vero cells in serum-free GMEM supplemented with antibiotics.

### Vero cell growth kinetics by RTCA and roller bottle systems

#### Small-scale Vero kinetics in the RTCA system

The RTCA system analyses and measures the electronic impedance in 16- or 96-well *E*-plates, which comprise microelectrode arrays at the bottom surface of each plate well. Vero cell suspension containing 1–5×10^4^ cells ml^−1^ was utilized for the seeding of the plates throughout the study, as described elsewhere [15]. In a 16-well *E*-plate, 100 µl Vero cells were added in duplicate wells with an additional 100 µl GMEM, bringing the total volume in each well to 200 µl. The *E*-plate was loaded onto the RTCA workstation (software 2.0.0.1301; ACEA Biosciences) at 37±2^ ^°C with a 5 % CO_2_ atmosphere. Cell growth status was monitored by recording the CI every hour for up to 200 h.

#### Pilot-scale Vero kinetics in the roller bottle system

A roller bottle was seeded with 20 ml of the same Vero suspension containing 1–5×10^4^ cells ml^−1^. The media was topped up with GMEM to 200 ml. Roller bottles were incubated at 37±2 °C, with cell growth status being monitored every 24 h for 200 h by cell counts using a TC10 automated cell counter (Bio-Rad) after staining with trypan blue 0.2 %.

#### Real-time comparison of CI with Vero viable cell count

Vero cell suspension containing 1×10^4^ cells ml^−1^ was utilized for the seeding of the plates. In a 16-well *E*-plate, 100 µl Vero cells were added in duplicate wells with an additional 100 µl GMEM, bringing the total volume in each well to 200 µl. The *E*-plate was loaded onto the RTCA workstation (software 2.0.0.1301; ACEA Biosciences) at 37±2^ ^°C with a 5 % CO_2_ atmosphere. Cell growth status was monitored by collecting the supernatant in each duplicate well daily for 8 days. Each day, the CI was recorded before samples were collected. Cell counts were conducted on the supernatant using a TC10 automated cell counter (Bio-Rad) after staining with trypan blue 0.2 %.

### Real-time RVFV clone 13 quantification using CI in the RTCA system and plaque-forming unit (p.f.u.) assay for the roller bottle system

#### Small-scale quantification in the RTCA system

Vero cells were used for RVFV clone 13 growth kinetics. A Vero cell suspension of 1–5×10^4^ cells ml^−1^ was used, 100 µl cell suspension was added per well. The RVFV clone 13 virus was serially diluted by mixing 500 µl virus with 4.5 ml serum-free GMEM. A 10-fold dilution series of RVFV clone 13 was prepared from 1×10^7^ to 1×10^1^ p.f.u. ml^−1^ (1 : 10–1 : 1 000 000) and 100 µl each dilution was added into an appropriate *E*-plate well in duplicate. The *E*-plate was incubated at 37±2^ ^°C with a 5 % CO_2_ atmosphere for 1 h before an extra 100 µl serum-free GMEM was added. The *E*-plate was loaded onto the RTCA system workstation at 37±2^ ^°C with a 5 % CO_2_ atmosphere for viral replication to occur, with CI value measured every hour for 120 h. CPE was reflected by a sudden decline in the CI value.

#### CIT_median/50_ (time to achieve the CI_median/50_) determination

CI values for each virus dilution utilized to infect cells in each well were determined for comparison with viral plaque assays. The maximum CI (CI_max_) and time for each RVFV clone 13 virus dilution were determined through the observation and determination of the CI_max_ value peak and time at the peak for each virus dilution. These measurements were utilized to determine the CI_median/50_ and CIT_median/50_. The determined CIT_median/50_ for each RVFV clone 13 virus dilution was plotted against the virus concentration obtained with the p.f.u. ml^−1^ assay. The correlation between the p.f.u. ml^−1^ assay quantification and RTCA CIT_median/50_ was verified using a linear regression analysis, where the linearity was estimated by analysis of R^2^ [10].

#### Pilot-scale quantification in the roller bottle system

Vero cells were grown to 100 % confluency in roller bottles before infecting with RVFV clone 13. Serial dilutions of RVFV clone 13 from 1×10^7^ to 1×10^1^ p.f.u. ml^−1^ were prepared and used to infect the roller bottle confluent Vero cells in triplicate for each dilution. Infected roller bottle cells were incubated at 37±2^ ^°C for viral replication to occur. Mock-infected cells with serum-free GMEM were used as controls in all experiments. Daily harvesting of the triplicate repeats of each dilution was conducted and the samples were pooled into one roller bottle. Harvesting was conducted from 24 to 120 h and viral plaque assays were conducted on harvested samples.

### Virus quantification by viral plaque assay

Infectious RVFV clone 13 viruses were quantified by viral plaque assays on Vero cell monolayers and results were expressed as log_10_ p.f.u. ml^−1^ [11]. Twenty-four hour Vero cell monolayers were prepared on 12-well tissue-culture treated plates. Serial 10-fold dilutions of the viral sample were prepared in serum-free GMEM. The medium from the 12-well monolayer plates was discarded, and working in triplicate wells from the lowest to the highest dilution, 0.5 ml RVFV clone 13 at appropriate dilutions was added to each well. Following an adsorption period of 1 h at 37±2^ ^°C with a 5 % CO_2_ atmosphere, 2 ml of 1 % (w/v) low-melting agarose (Laboratorois Conda) overlay was added. The plates were kept at 37±2^ ^°C for 5 days with a 5 % CO_2_ atmosphere before 1 ml of 1 % (v/v) neutral red stain (Merck Millipore) in GMEM was added to each well. The plates were incubated for an additional 24 h at 37±2 °C. Plaques were enumerated and the titre determined on day 6.

### Real-time comparison of CI and RVFV clone 13 virus titre

Vero cells were used for RVFV clone 13 growth kinetics. A Vero cell suspension of 1×10^4^ cells ml^−1^ was used; 100 µl of the cell suspension was added per well. The RVFV clone 13 virus was serially diluted by mixing 500 µl virus with 4.5 ml serum-free GMEM. A 10-fold dilution of RVFV clone 13 was prepared from 1×10^7^ to 1×10^6^ p.f.u. ml^−1^ (1 : 10) and 100 µl of the dilution was added into an *E*-plate. The *E*-plate was incubated at 37±2^ ^°C with a 5 % CO_2_ atmosphere for 1 h before an additional 100 µl serum-free GMEM was added. Virus replication was monitored by collecting the supernatant in each duplicate well daily for 8 days. Each day, the CI was recorded before samples were collected and viral plaque assays were conducted on harvested samples.

### Statistical analysis

TIBCO Statistica version 13.0 software was utilized for all statistical analyses conducted in this study. Statistical analyses were conducted in order to determine a correlation between the RTCA and roller bottle systems. Spearman’s rank correlation analysis [21] was performed in order to assess the correlation between (i) CI and viable Vero cell counts, and (ii) RVFV clone 13 virus titre and CI.

## Results

### Vero cell growth kinetics by RTCA and roller bottle systems

**Fig. 1.**
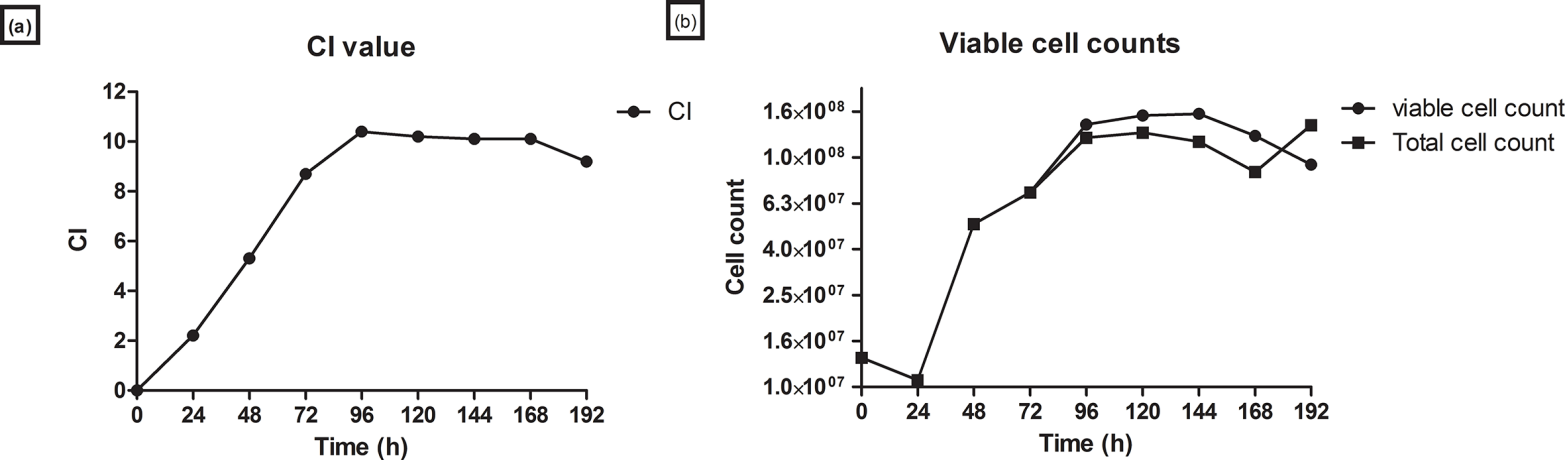
Vero host cell line growth kinetics. (a) Real-time growth kinetics of Vero host cell line on the RTCA system. A measure of cell–cell attachment is given by CI. (b) Growth kinetics of Vero host cell line on the roller bottle system.

Vero host cell growth kinetics were assessed on both the RTCA and roller bottle systems. The CI was monitored on the RTCA system to indicate various growth phases of the Vero cell line. An increase in CI from 0 to 2 was observed in the first 48 h. The Vero exponential phase was between 48 and 100 h, where the CI value increased rapidly. The stationary phase was observed from approximately 100 to 168 h, before the death phase. There was a decrease in the CI value from 168 to 196 h (Fig. 1a). In the roller bottle system, viable cell counts were monitored to indicate the various growth phases of the Vero cell line. Viable cell count increased from 2×10^9^ to 1.02×10^10^ between 48 and 100 h, before dropping to 8×10^9^ between 120 and 196 h. Similarly to observations in the RTCA system, the Vero exponential phase was between 48 h to approximately 100 h in the roller bottles. The stationary phase was observed from 100 h to approximately 120 h, before the death phase was observed (Fig. 1b). The CI on the RTCA system for the Vero cells increased to a maximum of 10. The maximum viable cell count in roller bottles was 1.2×10^8^ cells ml^−1^. There was a correlation in the RTCA and roller bottle systems in terms of the Vero growth curves and the time taken to reach maximum cell density as reflected by CI value and viable cell count.

### Real-time comparison of CI with Vero viable cell count

**Fig. 2.**
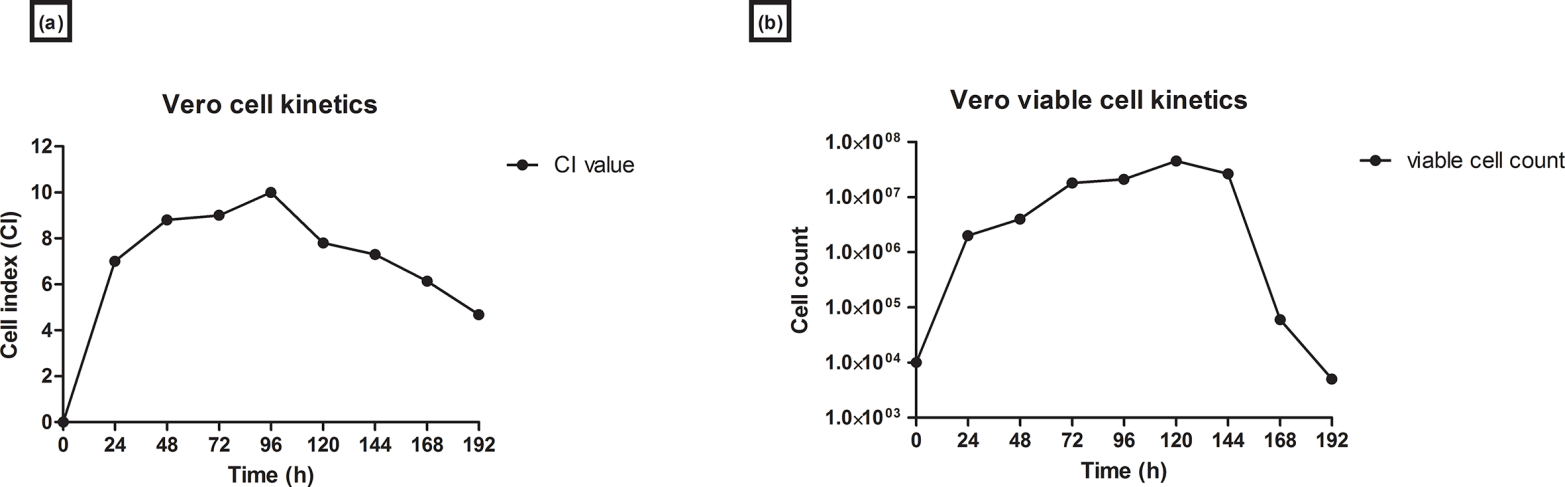
Vero host cell line growth kinetics. (a) Real-time growth kinetics of the Vero host cell line on the RTCA system. A measure of cell–cell attachment is given by CI. (b) Growth kinetics of the Vero host cell line from supernatant samples collected daily from *E*-plate wells.

Vero host cell growth kinetics were assessed with both the RTCA CI readings and the supernatant collected from the *E*-plate wells. The supernatant samples were stained with trypan blue 0.2 % and cell counts obtained. Vero cell growth phases were observed and compared for the CI and supernatant samples of the Vero cell line. An increase in CI from 0 to 8 was observed in the first 48 h. Viable cell counts with trypan blue increased from 1×10^4^ to 4×10^6^ cells ml^−1^. The exponential phase of the cells was observed from 24 to 96 h, where both the CI and the cell counts were observed to increase. The stationary phase was observed from approximately 100 to 144 h, before the death phase. There was a decrease in the CI value and cell counts from 144 to 192 h (Fig. 2). The CI on the RTCA system for the Vero cells increased to a maximum of 10. The maximum viable cell count in roller bottles was 4.7×10^7^ cells ml^−1^. Comparative data was obtained in the RTCA and roller bottle systems for Vero growth curves and the time taken to reach maximum cell density as reflected by CI value and viable cell count, respectively.

### Statistical analysis using Spearman’s rank correlation – CI and Vero viable cell count

**Fig. 3.**
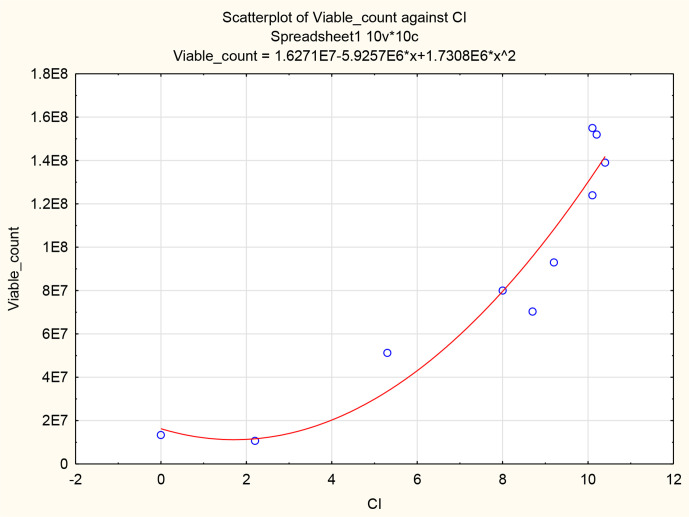
A scatterplot of a Spearman’s rank correlation analysis of CI values obtained from the RTCA system and viable cell counts obtained from the roller bottle system as variables.

Spearman’s correlation statistical analysis was conducted in order to determine the strength of the correlation between the CI from the RTCA system and viable cell counts obtained from the roller bottle system as variables. The CI and viable cell count scatterplot indicates a monotonic relationship. The increase in Vero cell viability is indicated by an increase in CI values (Fig. 3). The strength of the monotonic relation of CI and viable cell counts was determined with the Spearman’s correlation coefficient (r_s_). The CI to viable cell count r_s_ was 0.911, suggesting the relationship is very strong.

### Real-time RVFV clone 13 quantification using CI in the RTCA system

**Fig. 4.**
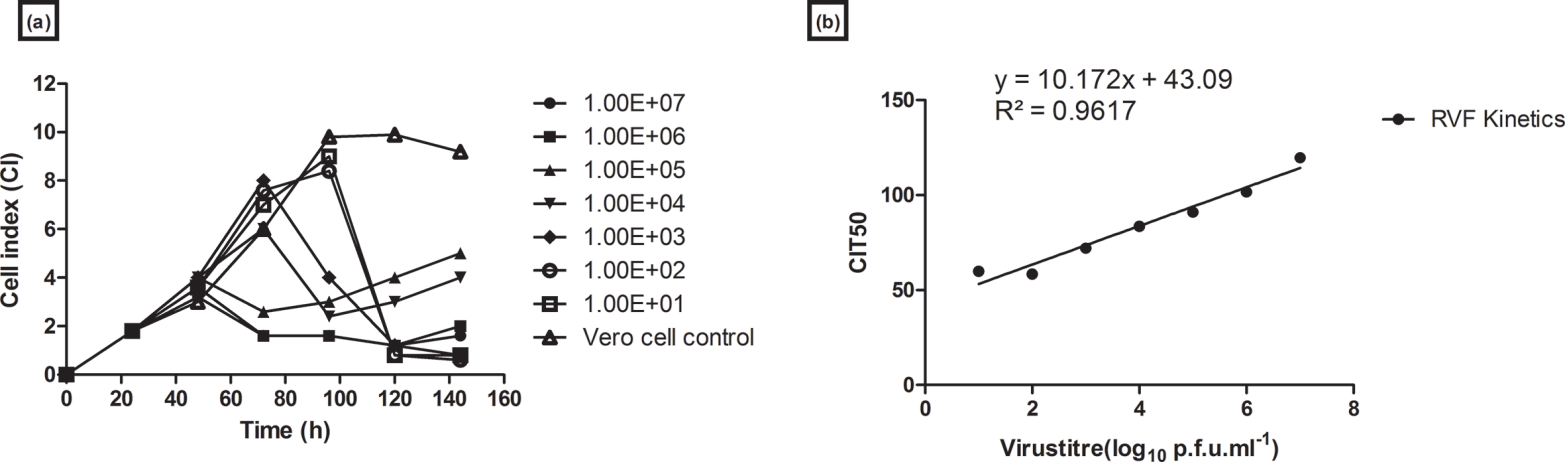
Real-time viral monitoring of RVFV clone 13 virus CPE on Vero host cells in the RTCA system. (a) CI post-infection for *E*-plate wells inoculated with various serial dilutions of RVFV clone 13. The curve indicates the mean of duplicate wells. (b) Regression line of RVFV clone 13 CPE on Vero cells from the RTCA system data.

RVFV clone 13 virus titrations were conducted in the RTCA system in order to investigate the use of the system as an alternative to the current p.f.u. quantification assay. The effects of viral replication in the form of CPE were observed as a drop in the CI value. No CPE was observed in Vero cell control samples as no RVFV clone 13 virus had been added to the samples (Fig. 4a). An increase in CPE formation time was observed with samples of high RVFV clone 13 concentration. Vero cells infected with 10^7^–10^5^ p.f.u. ml^−1^ virus dilution had CPE formation at 50 h post-infection. The CI for these samples was observed to be at 3.8 at 48 h and began dropping to 2 at 50 h, before plateauing to below 1 after 60 h. Vero cells infected with 10^4^ and 10^3^ p.f.u. ml^−1^ virus dilutions had CPE formation at 60 h post-infection. The CI for these samples was observed to be at six and eight for the dilutions, respectively, at 60 h and began dropping to two for the 10^4^ p.f.u. ml^−1^ virus dilution at 100 h and one for the 10^3^ p.f.u. ml^−1^ virus dilution at 120 h. Vero cells infected with 10^2^ and 10^1^ p.f.u. ml^−1^ virus dilutions were observed to have CPE formation at 100 h post-infection. A CI of eight was observed for these sample dilutions at 100 h and began dropping to below one at 120 h (Fig. 4a). A regression line of RVFV clone 13 CPE on Vero cells from RTCA system data was generated and resulted in a R^2^ of 0.96, suggesting a positive correlation between CI and RVFV clone 13 titre (Fig. 4b). CIT50 represents the time it took the CI at each dilution to decrease by 50 % after virus infection.

### Viral quantification of RVFV clone 13 in the roller bottle system by p.f.u. assay

**Fig. 5.**
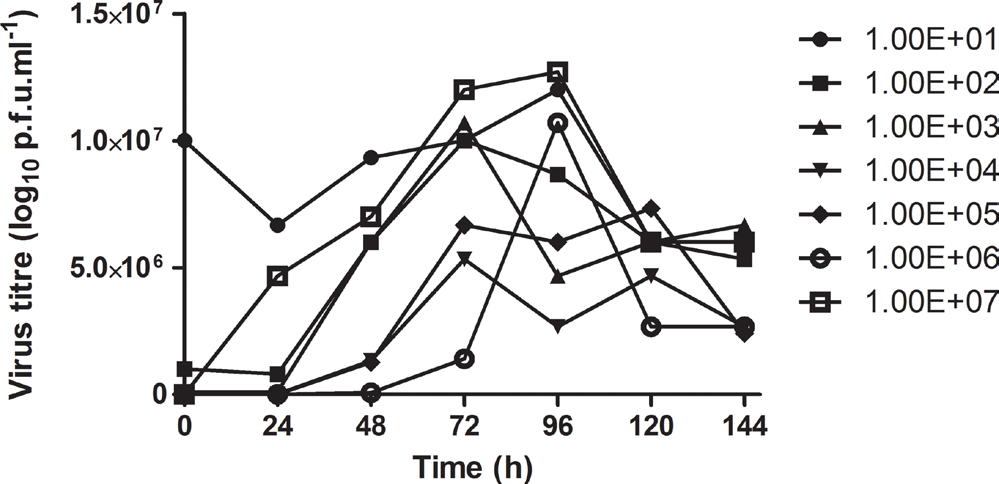
RVFV clone 13 viral plaque assay quantification of RVFV cultures in the Vero roller bottle system.

Viral plaque assays were conducted for RVFV clone 13 virus samples propagated in roller bottles. These results were compared to the RTCA quantification results. A decrease in CPE formation time was observed with samples of high RVFV clone 13 concentration. A CPE for the 1×10^7^ virus dilution sample was observed from 48 to 96 h. RVFV clone 13 titre was observed to increase from 1.0×10^1^ to 1.2×10^7^ p.f.u. ml^−1^ at 48 and 96 h, respectively. CPEs for the 1×10^6^ and 1×10^5^ virus dilution samples were observed from 24 to 96 h, with virus titre observed to increase from 5.0×10^6^ to 1.0×10^7^ p.f.u. ml^−1^ at 24 and 96 h, respectively. RVFV clone 13 titres were then observed to drop to 6.0×10^6^ p.f.u. ml^−1^ at 120 h for these virus dilution samples. Samples with 1×10^4^ and 1×10^3^ virus dilutions had CPE from 48 to 72 h. RVFV titre for the 1×10^4^ dilution was observed to increase from 5.0×10^6^ to 6.0×10^6^ p.f.u. ml^−1^ at 24 and 72 h, respectively. Titres for the 1×10^3^ dilution were observed to increase from 5.0×10^6^ to 6.0×10^6^ p.f.u. ml^−1^ at 24 and 72 h, respectively. No significant viable RVFV clone 13 titre increase was observed at 120 h for either dilution sample. CPE for the 1×10^2^ dilution sample was observed between 72 and 96 h. An increase in RVFV titre from 5.0×10^6^ p.f.u. ml^−1^ at 24 h to 1.0×10^7^ p.f.u. ml^−1^ at 100 h was observed before the titre dropped to 2.0×10^6^ p.f.u. ml^−1^ at 120 h. CPE for the 1×10^1^ virus dilution sample was observed from 24 to 96 h. Titre was observed to increase from 5.0×10^6^ p.f.u. ml^−1^ at 24 h to a high titre of 1.2×10^7^ p.f.u. ml^−1^ at 96 h, before the titre was observed to drop to 5.0×10^6^ p.f.u. ml^−1^ at 120 h (Fig. 5). When the plaque forming assay results were compared to results obtained from the RTCA system, the log_10_ titres (p.f.u. ml^−1^) were comparable for various dilutions. The high 1×10^7^ p.f.u. ml^−1^ titre on the RTCA linear regression graph for the 1×10^7^ p.f.u. ml^−1^ sample was at approximately 100 h as compared to the 96 h in the roller bottle system. Furthermore, the 1×10^1^ sample reached 1×10^7^ p.f.u. ml^−1^ at 96 h, whilst on the RTCA linear regression graph it was 100 h. These results were comparable between the RTCA CIT_median/50_ and roller bottle p.f.u. ml^−1^ data (Fig. 4b).

### Real-time comparison of CI and RVFV clone 13 virus titre

**Fig. 6.**
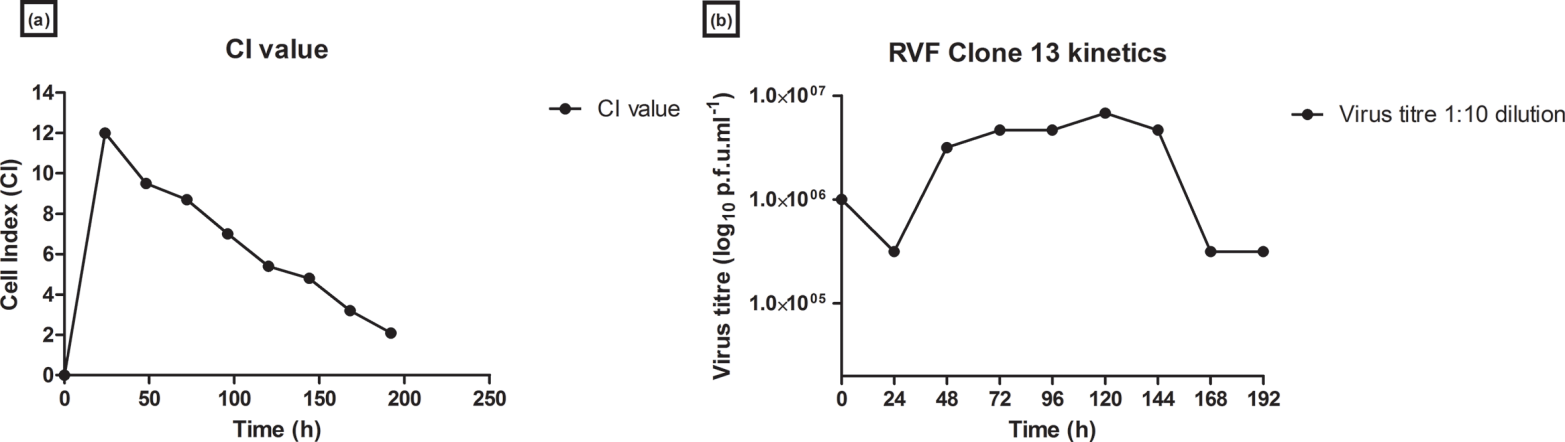
Real-time viral monitoring of RVFV clone 13 virus CPE on Vero host cells on the RTCA system. (a) CI post-infection for *E*-plate wells inoculated with the 1 : 10 (1×10^6^ p.f.u. ml^−1^) dilution of RVFV clone 13. The curve indicates the mean of duplicate wells. (b) RVFV clone 13 virus quantification of RVFV cultures collected from *E*-plate well supernatant daily for 8 days.

RVFV clone 13 replication kinetics analysis was conducted on both the RTCA CI readings and the supernatant collected from the *E*-plate wells. Viral replication in the form of CPE was observed as a drop in the CI value. No CPE was observed from 0 to 24 h, as the CI was shown to increase from 0 to 12. At 24 h, CPE was initiated as a drop in CI is observed until 192 h (Fig. 6a). Viral plaque assays were conducted for RVFV clone 13 virus (1 : 10) supernatant samples collected from *E*-plate wells. These results were compared to the CI values obtained in Fig. 6(a). CPE for the virus dilution sample was observed from 24 to 96 h. Titres were observed to increase from 3.16×10^5^ p.f.u. ml^−1^ at 24 h to a high titre of 6.8×10^6^ p.f.u. ml^−1^ at 120 h, before titres were observed to drop to 3.16×10^5^ p.f.u. ml^−1^ at 192 h (Fig. 6b). When the plaque forming assay results were compared to results obtained from the CI values, similarities were observed.

### Statistical analysis using Spearman’s rank correlation – CI and virus titre

**Fig. 7.**
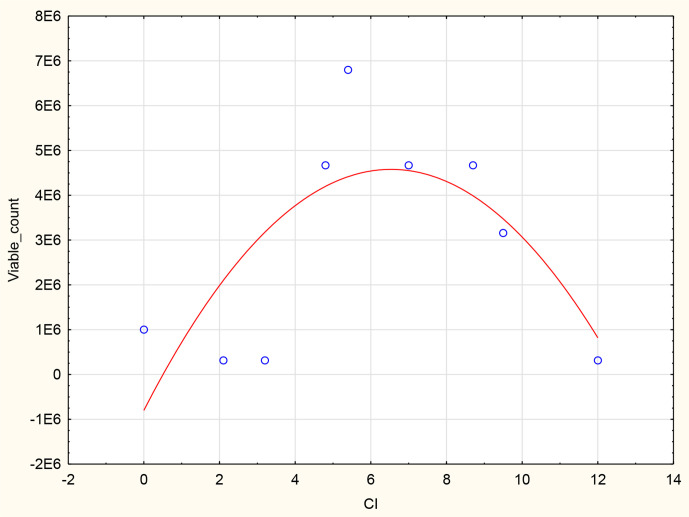
A scatterplot of a Spearman’s rank correlation analysis of CI values obtained from the RTCA system and RVFV clone 13 titre obtained from the roller bottle system as variables.

Spearman’s correlation statistical analysis was conducted in order to determine the strength of the correlation between the CI from the RTCA system and RVFV clone 13 antigen titre obtained from the roller bottle system as variables. The CI and viable cell count scatterplot indicates a monotonic inverse relationship (non-monotonic). The increase in RVFV clone 13 virus titres is indicated by a decrease in CI values (Fig. 7). The strength of this relation was determined with the Spearman’s correlation coefficient (r_s_). The CI to viable cell count r_s_ was 0.189, confirming the non-monotonic relationship.

## Discussion

This study focused on investigating the utilization of the RTCA system as an alternative quantification method to the p.f.u. assay and for real time study of Vero cell growth and RVFV clone 13 cell infection. Vero cell growth kinetics results on the RTCA and roller bottle systems were comparable. The Spearman’s rank correlation analysis indicated a strong monotonic relationship between Vero cells and the CI variables as an increase in cell viability correlated to an increased CI value. This suggests the utilization of the RTCA system as an alternative to the time-consuming roller bottle system reaches similar outputs. Not only does the RTCA system reduce workload and contaminations in terms of laboratory sample handling, but it also allows for label-free observation of viable cells utilizing less media and cells as compared to the roller bottle system. Furthermore, the RTCA system allows for real-time analysis of multiple samples as compared to the limiting roller bottle system, where only one sample can be analysed at a time. The RTCA system was observed as an efficient, fast and superior cell analysis method to the 3-(4,5-dimethylthiazol-2-yl)−2,5-diphenyltetrazolium bromide (MTT) cell proliferation and PrestoBlue assays for various cell cultures in the testing of water contaminations [22].

The viral plaque assay quantification of RVFV clone 13 in the roller bottle system and the CIT50 of the antigen in the RTCA system were shown to be comparable. CPE was monitored by declines in the CI value of RVFV clone 13-infected Vero cells. The Spearman’s rank correlation analysis indicated a strong non-monotonic relationship between RVFV clone 13 infected cells and CI values. An increase in RVFV clone 13 viral titre correlated with a decrease in CI value at the time points investigated. This phenomenon was similarly demonstrated in H1N1, where a decline in CI value for the RTCA system was shown to be as a result of cell death due to virus replication [23]. In viruses where CPE is not visible, the RTCA system showed significant changes in CI values, indicating that the system may be utilized for a variety of viruses [24]. In this study, a decline in CI value was observed for all Vero cultures infected with the antigen at various dilutions over time. An increase in CPE formation time was observed with an increase in dilution factor as found in a similar study where the RTCA system was successfully evaluated for its utilization in quantification of monovalent and tetravalent chimeric yellow fever dengue vaccine virus as an alternative to the cell culture infectious dose 50 % (CCID_50_) method [10]. The RTCA system was identified as a robust and reproducible assay system for quantification of immunotherapy reagents, which has the ability to reduce the use of fluorescent and radioactive labels at research and development levels [25]. The RTCA assay was also proposed for utilization as a quality-control potency assay in manufacturing and large screening experiments [25]. In addition to the findings, the RTCA system is a platform that can be utilized in improving the animal-health sectors. A multiple sample screening ability of the RTCA system allows more laboratory tests to be conducted and less subjective results to be obtained by researchers; thus, eliminating time taken for experimental repeats.

This work is the first report, to the best of our knowledge, on the application of the RTCA system to RVFV clone 13 virus infection kinetics and its comparison to a roller bottle system. Cell culture kinetics may also be performed on the RTCA system with a high number of replicates; thus, decreasing manual workload in laboratories when utilizing roller bottle systems. Furthermore, the RTCA system offers high-throughput screening through reducing the handling of samples.

This study has demonstrated that RTCA is an alternative method to the time-consuming roller bottle and viral plaque assays for conducting Vero tissue-culture kinetics and RVFV clone 13 virus quantification titrations, respectively. The RTCA system can be used for rapid optimization of processes for vaccine production as shown for the RVFV clone 13 virus in this study.
